# Titanium Corrosion Mechanisms in the Oral Environment: A Retrieval Study

**DOI:** 10.3390/ma6115258

**Published:** 2013-11-15

**Authors:** Danieli C. Rodrigues, Pilar Valderrama, Thomas G. Wilson, Kelli Palmer, Anie Thomas, Sathyanarayanan Sridhar, Arvind Adapalli, Maria Burbano, Chandur Wadhwani

**Affiliations:** 1 Department of Bioengineering, University of Texas at Dallas, 800 W Campbell Rd, Richardson, TX 75080, USA; E-Mails: axt012600@utdallas.edu (A.T.); sxs125031@utdallas.edu (S.S.); apa103020@utdallas.edu (A.A.); mxb121131@utdallas.edu (M.B.); 2 Department of Periodontics, Texas A&M University Baylor College of Dentistry, 3302 Gaston Av, Dallas, TX 75246, USA; E-Mail: valderrama@bcd.tamhsc.edu; 3 Private Practice of Periodontics, 5465 Blair Road suite 200, Dallas, TX 75231, USA; E-Mail: tom@northdallasdh.com; 4 Department of Molecular and Cell Biology, University of Texas at Dallas, 800 W Campbell Rd, Richardson, TX 75080, USA; E-Mail: kelli.palmer@utdallas.edu; 5 Department of Restorative Dentistry, University of Washington, 1959 NE Pacific Street, Seattle, WA 98195, USA; E-Mail: cpkw1@live.com

**Keywords:** titanium, dental implants, corrosion, peri-implantitis

## Abstract

Corrosion of titanium dental implants has been associated with implant failure and is considered one of the triggering factors for peri-implantitis. This corrosion is concerning, because a large amount of metal ions and debris are generated in this process, the accumulation of which may lead to adverse tissue reactions *in vivo*. The goal of this study is to investigate the mechanisms for implant degradation by evaluating the surface of five titanium dental implants retrieved due to peri-implantitis. The results demonstrated that all the implants were subjected to very acidic environments, which, in combination with normal implant loading, led to cases of severe implant discoloration, pitting attack, cracking and fretting-crevice corrosion. The results suggest that acidic environments induced by bacterial biofilms and/or inflammatory processes may trigger oxidation of the surface of titanium dental implants. The corrosive process can lead to permanent breakdown of the oxide film, which, besides releasing metal ions and debris *in vivo*, may also hinder re-integration of the implant surface with surrounding bone.

## 1. Introduction

Titanium (Ti) and its alloys are broadly used in the design of dental and orthopedic implants, due to a combination of attractive properties that include high corrosion resistance, biocompatibility, re-passivation and adequate mechanical properties. The corrosion resistance of Ti and its alloys is a result of the material’s ability to spontaneously form passive oxide films (TiO_2_) when in contact with oxygen [1]. Ti oxide is a stable and dense layer, which acts as a protective barrier to continued metallic oxidation. In the event of damage, TiO_2_ has the ability to spontaneously reform under normal physiological conditions. However, events, such as abnormal cyclic loads, implant micromotion, acidic environments and their conjoint effects, can result in permanent breakdown of the oxide film, which may consequently lead to exposure of the bulk metal to an electrolyte.

Because the oral environment will subject titanium to conditions of varying pH, due to inflammatory or other processes that can turn the medium acidic, active dissolution of metal ions can occur upon exposure of the bulk metal [2,3,4]. A few studies have reported cases of severe corrosion of titanium dental implants as being the cause for implantation failure [5] or one of the triggering factors for peri-implantitis [6]. Corrosion of dental implants is concerning, because a large amount of metal ions and debris are generated in this process, of which accumulation may lead to adverse tissue reactions in the oral environment [6]. In summary, the main events linked to Ti implant degradation in the oral environment seem to be related to: (1) electrochemical factors, acidity caused by the presence of inflammatory processes, oral bacteria or the use of solutions that can attack the surface of the implant; (2) mechanical factors, induced by mechanical loads that can lead to fretting and excessive wear of the surface; and (3) synergistic action of electrochemical and mechanical factors (tribocorrosion).

Cases of severe corrosion in Ti modular junctions of total hip implants have been reported in the orthopedics literature, which illustrate the susceptibility of this material to degradation *in vivo* [7,8]. These cases have been often associated with mechanisms of fretting-crevice corrosion induced by implant modularity [9]. This particular corrosion mechanism is triggered by stagnant acidic body fluid entrapped in crevices of mating connections that undergo micromotion during normal loading [9]. Crevice corrosion is, therefore, a localized form of corrosion attack at contacting interfaces, such as metal-on-metal and, potentially, metal-on-bone, with restricted ingress and egress of fluid and depletion of oxygen. In these restricted contacting areas, physiological fluid will become acidic, due to the presence of free H ions in the medium. When H ions are free to interact with electrons, the pH drops significantly, and active metal dissolution can occur. Other events, such as pitting attack, surface delamination and etching of Ti implant surfaces, give indications of degradation induced by a very acidic environment.

It is known that normal oral bacteria and other inflammatory processes may induce oxide film disruption and interrupt osseointegration [10]. Bacterial colonization on these dental implant surfaces occurs almost immediately after implantation, and colonization by diverse microorganisms results in formation of dental biofilms [11,12,13]. Although the rate of failure of dental implants caused by corrosion associated with bacterial biofilm is unknown or less studied, it is hypothesized that the adherence of bacteria and its sub-products could disrupt the passivity of Ti surfaces [5]. This is an important observation given that reduction of Ti oxide layers will prevent incorporation of calcium ions, which will hinder re-integration with the implant surface. Bacterial colonization on the surface of a Ti implant may lead to two events: (1) Bacteria will significantly reduce the pH of the oral environment by the production of organic acids during sugar catabolism, which will initiate metal dissolution. This low pH may create a favorable environment for corrosion to take place. It has been shown *in vitro* that corrosion of Ti grade 2 was higher in saliva containing *Escherichia coli* lipopolysaccharide (LPS) and at low pH levels [3]. Suito *et al.* [14] demonstrated in an immersion study of Ti in simulated body fluid of varying pH that the lower the pH and the longer the immersion time, the greater the amount of Ti ions released. The same behavior was observed in the presence of mechanical stimulus during immersion and when in contact with a dissimilar metal. Another study demonstrated opposite results, with degradation peaking at near neutral pH values in the presence of motion [2]. This controversy indicates that the mechanism behind the elution of Ti *in vivo* remains unclear. These corrosion products can induce inflammation and bone loss, leading to osseointegration instability [15]; (2) another hypothesis is that the creation or deposition of biofilm on the dental implant surface leads to differential oxygen exposure on the implant surface. The less aerated zones will act as the anode and will undergo crevice corrosion, releasing metal ions into saliva. This will further favor a corrosive environment for the dental implant, by the combination of metal ions released, the end products of bacteria and chloride ions present in saliva [5].

Therefore, a drop in pH due to the presence of bacteria, bacterial biofilm, which may create a crevice environment, and other inflammatory processes (e.g., peri-implantitis, peri-mucositis) may create the ideal conditions for Ti oxidation. The goal of this study is to evaluate the surface of retrieved titanium dental implants due to peri-implantitis. Five implants showing particular corrosion features were characterized using different microscopy techniques. Characterization of these surfaces can help to clarify the mechanisms and dynamics behind the elution of Ti in the body, which remains unclear up to this date [14].

## 2. Materials and Methods

Implants retrieved from five patients exhibiting signs of adverse tissue reaction were investigated. All the specimens were obtained from patients attending a private periodontics clinic. Five patients with a history of peri-implantitis provided consent according to the guidelines of the Helsinki Declaration to donate their retrieved implants for research. Once the implants were retrieved and stored, they had no identifiers that could be linked to the patient who donated the implant. Therefore, information like time of service *in vivo* was not available, with the exception of one implant, for which longevity was known. Implant characteristics (type and size) are summarized in Table 1. All the implants had different designs and sizes. The specimens were subjected to cleaning followed by autoclave sterilization prior to analysis.

Upon receipt of implants, a visual inspection was first performed to detect particular areas of interest on the implants, gross features and to verify the severity of corrosion present (discoloration, cracking and metallic debris). Specimen condition as received was recorded for all the specimens. One non-implanted specimen was used as control for comparison with the surface of each of the five specimens in analysis. The implants were then analyzed with low (0×–50×) and high (100×–1000×) magnification digital microscopy (Keyence VHX-2000, Itasca, IL, USA) for identification of surface features and failure mechanisms. The same microscopy technique was used to verify the depth of surface features, such as pits and scratches, using 3D depth features. Areas of interest were marked for further analysis using scanning electron microscopy (SEM, JEOL, JSM-6010, Peabody, MA, USA). The SEM was equipped with an energy dispersive X-ray spectrometer (EDS), which provided the composition of the sample’s bulk and oxide film. EDS analysis revealed the composition of areas with signs of corrosion or biological deposition.

After surface characterization, all the specimens were subjected to a secondary cleaning protocol for complete removal of biological deposits that could have been covering other important surface features. The implants were first cleaned in an aqueous solution with soap powder, followed by cleaning in distilled water and, finally, 70% ethanol. For cleaning, specimens were fully immersed in each of the solutions and subjected to sonication for 1 hour. Microscopy and EDS was then repeated for confirmation of the observed features.

**Table 1:** Implant identification.

Implant ID	Size (diameter × length)	Implantation length
Control	ϕ 4.1 mm RN*	0
	SLA 12mm**	
	tapered effect implant	
Implant #1	4.8 mm	unknown
	6 mm	
Implant #2	3.3 mm	unknown
	10 mm	
Implant #3	4.1 mm	unknown
	8 mm	
Implant #4	4.8 mm	4 weeks
	10 mm	
Implant #5	4.8 mm	unknown
	10 mm	

*RN: regular neck; **SLA: sand blasted, large grit, acid etched surface.

## 3. Results

Analysis of the surface of the implants using the three microscopy techniques described revealed several common features and failure mechanisms among the specimens. A few of the specimens still had bone integrated with the rough interfaces of the implants. The 3D microscopic analysis showed evidence of severe corrosion and bulk exposure (post-cleaning). SEM and EDS provided information on the surface morphology and bulk structure.

**Figure 1:**
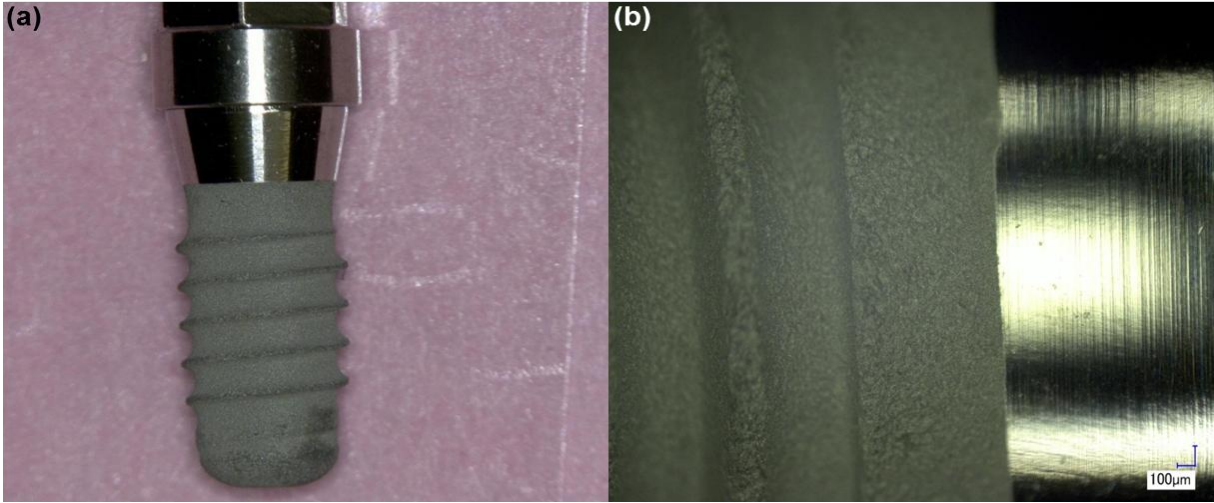
Control implant. (**a**) Low magnification overview of the surface of the implant; and (**b**) higher magnification showing surface condition of the smooth and rough interfaces of the implant.

Analysis of the control implant (Figure 1) demonstrated that the surface of the implant was clean of deposits and no particular features or deformation were noted in any of the components of the implant. Compositional analysis demonstrated that the control specimen was rich in Ti, for which concentration varied from 87% to 92% from the rough to the smooth interfaces of the implant, respectively. Low concentrations of oxygen were detected with the control specimen (3%–5%). This low oxygen concentration is probably a result of the etching process employed on the implant surface, which can lead to the replacement of oxygen by titanium hydrides [16].

**Figure 2:**
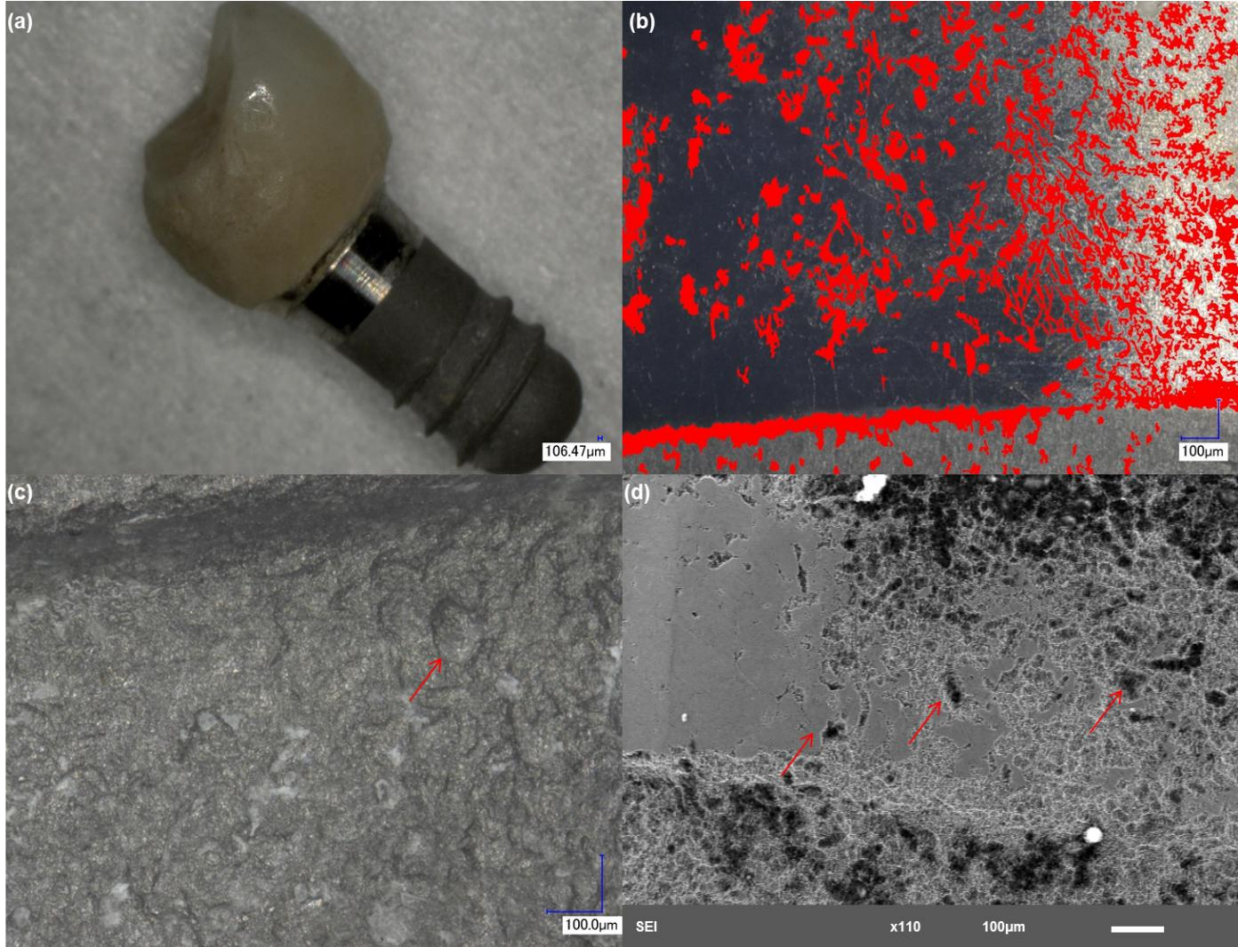
Structure of implant 1. (**a**) Low magnification showing overall features of the implant; (**b**) the smooth interfaces of the abutment showed a severe degree of pitting attack and scratching. The red areas highlight the pits present on the surface; (**c**) implant rough interface demonstrating pitting attack and deformities; and (**d**) higher magnification of an area with pitting attack and delamination of the top surface.

Evaluation of the surface of implant 1 (Figure 2) revealed deformities both in the abutment and in the rough interfaces of the specimen. A severe degree of scratching and pitting attack (Figure 2b) can be appreciated in the abutment of the implant. Pitting attack was also predominantly found in the smooth collar of the implant. The condition of the abutment surface points to a mechanism of fretting-crevice corrosion. Analysis of the rough interfaces of the implant showed the presence of pits and deformities of the top surface (Figure 2c,d) indicating a high degree of wear. The EDS results indicate bulk exposure of titanium (Ti rich) with high concentration of corrosion and biological products on the surface, such as carbon (C), nitrogen (N) and phosphorous (P) (Table 2). EDS analysis also showed evidence of zirconium (Zr), calcium (Ca) and trace concentrations of sulfur (S) on the surface of this implant.

**Figure 3:**
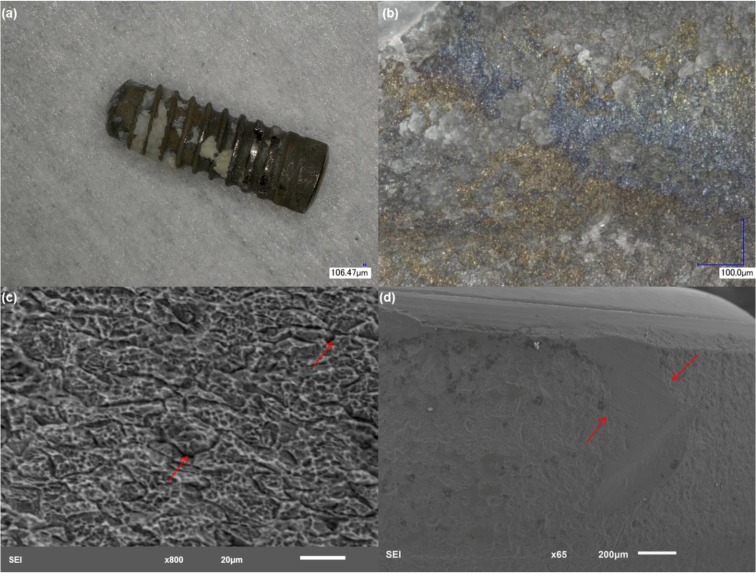
Structure of implant 2. (**a**) Low magnification showing the gross features of the implant; (**b**) discoloration is evident at a higher magnification. The violet and yellow discoloration indicates oxidation of Ti; (**c**) implant rough interface demonstrating severe cracking, which probably led to bulk exposure; and (**d**) higher magnification with removal of the top layers of the metal (arrows) in the top region of the implant.

Figure 3 shows the surface condition of implant 2. It is clear from microscopic analysis that this implant was exposed to a very acidic environment, which triggered electrochemical corrosion. This is evident from the violet and yellow discoloration of the rough interfaces (Figure 3b). High magnification analysis of the discolored interfaces revealed deformities with pitting attack and cracking of the bulk (Figure 3b,c). Pitting was mostly apparent in areas with the characteristic discoloration. The cracking pattern seems to have been nucleated inside pits with little branching. The top areas of the abutment showed delamination of the surface (Figure 3d, arrows), which exposed the bulk to accelerated dissolution. EDS of the areas shown in Figure 3 demonstrated high concentrations of Ti (up to 80%), which confirmed bulk exposure. These areas showed a depletion of oxygen (O). Other elements characteristic of corrosion products were present (C, N, P) similar to implant 1. Trace concentrations of S were also detected with this specimen.

**Figure 4:**
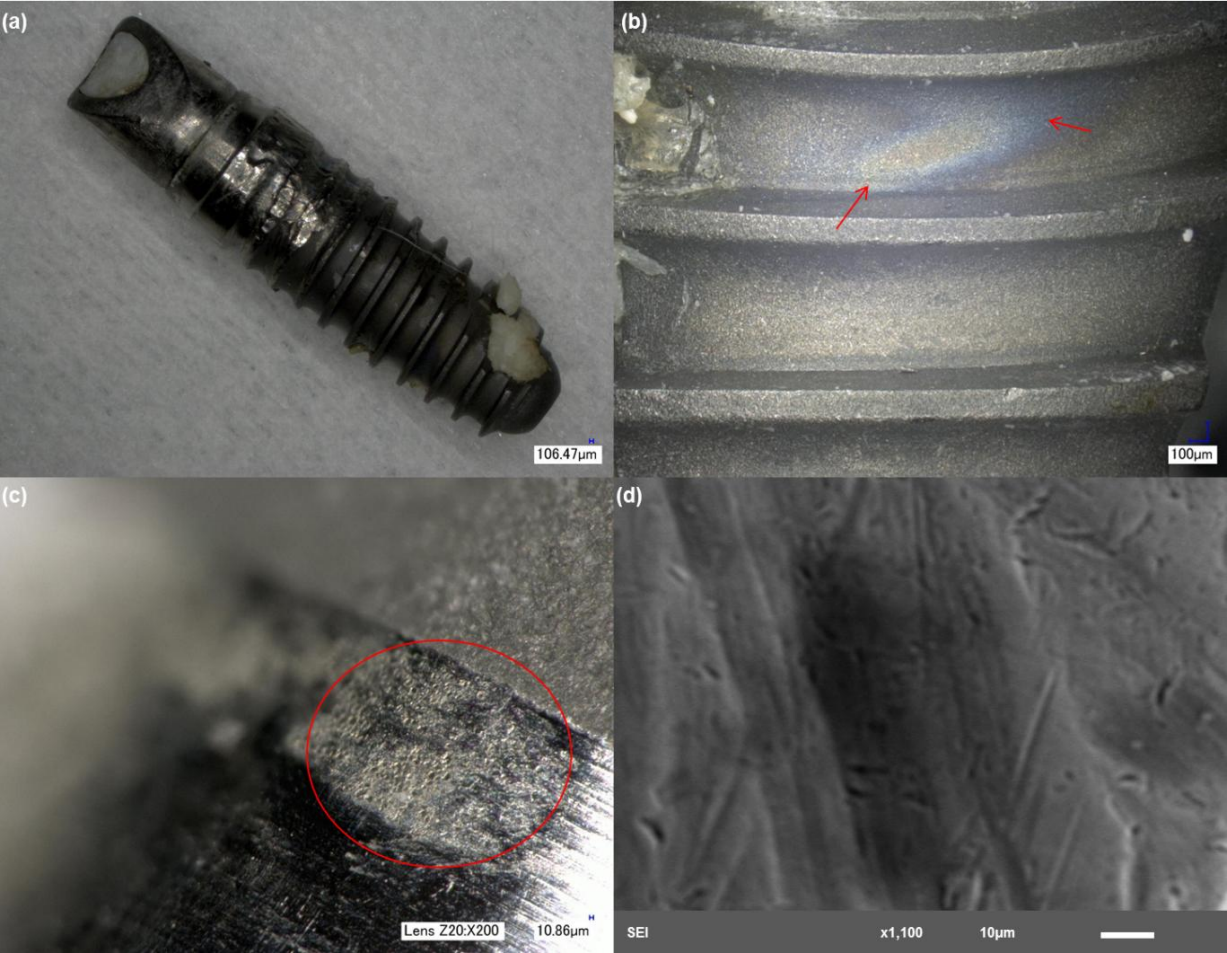
Structure of implant 3. (**a**) Low magnification showing the gross features of the implant and bone attachment to the bottom part of the surface; (**b**) discoloration is also evident in this example; (**c**) severe pitting attack on the top interface of the implant in the abutment region; and (**d**) higher magnification of an area of the abutment with scratching.

Evaluation of the surface of implant 3 (Figure 4) showed similar patterns in comparison to implant 2. Optical microscopy demonstrated similar discoloration (Figure 4b), indicating the presence of a very acidic environment surrounding the implant. The rough interfaces of the implant also exhibited pitting attack, deformation and scratches (Figure 4c). Analysis of the abutment showed severe scratching and small pits (Figure 4d), indicating that this area of the implant was subjected to a mechanism of fretting-crevice corrosion. EDS analysis of both the abutment and rough interfaces of this implant provided the presence of a high percentage of titanium (~50%–60%) and considerable percentage of aluminum (5%–10%) and vanadium (2%–4%). These percentages indicate that this implant in particular was made with Ti6Al4V alloy.

**Figure 5:**
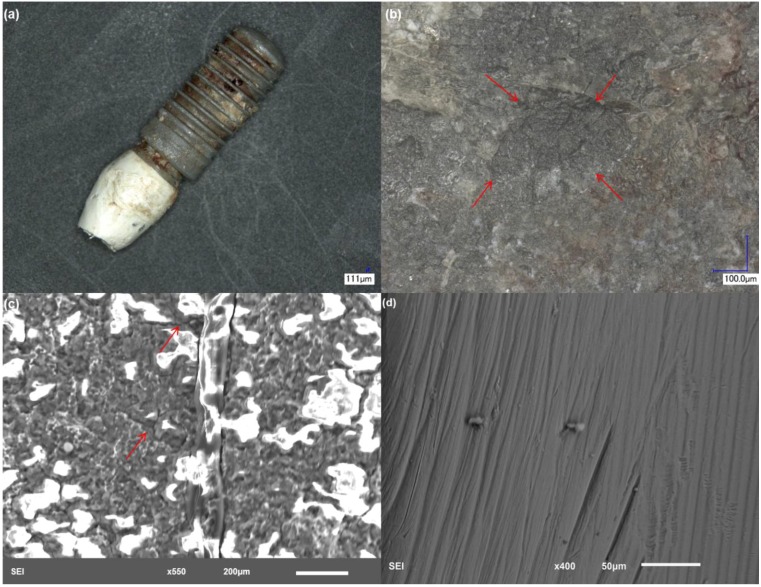
Structure of implant 4. (**a**) Low magnification showing the gross features of the implant and bone attachment to the bottom part of the surface; (**b**) discoloration is also evident in this example with severely deformed areas; (**c**) implant rough interface with evidence of crack development, which was filled with biological material; and (**d**) higher magnification of an exposed area of the abutment with scratching.

Implant 4 also showed severe deformation of the rough interfaces (Figure 5), exhibiting the characteristic discoloration surrounded by pits and areas with surface delamination (Figure 5b). Analysis of the smooth collar areas surrounding the abutment and areas with exposure of the bulk revealed the presence of a high percentage of titanium (~65%–70%) and a considerable percentage of aluminum (5%–10%) and niobium (~5%). Figure 5c shows the presence of a crack running through the rough surface. Branching of the crack can be observed in higher magnification. Crack branching is characteristic of stress corrosion cracking. Tracking the propagation of the branches was difficult, due to the presence of biological materials that resulted in bright/charged areas. Furthermore, the EDS of this region showed a very small percentage of Ti (~12%), which resulted from biological matter filling up the crack space, which can be confirmed in the EDS results from the increase in C, O and P levels. Similar to implants 2 and 3, the smooth collar area showed pitting attack and scratching. Because the crown was still in place with this particular sample, it was not possible to evaluate the entire surface of the abutment. This particular sample exhibited niobium in its composition, indicating that the implant was made of TiNbAl alloy.

**Figure 6:**
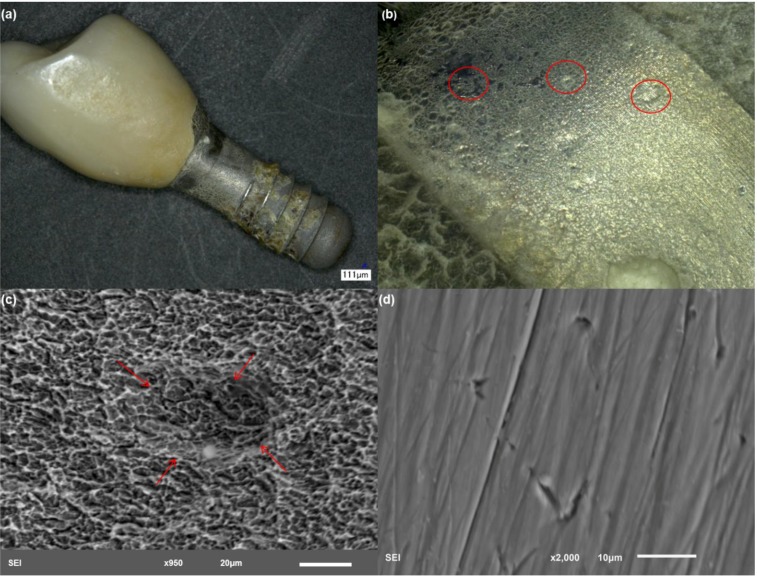
Structure of implant 5. (**a**) Low magnification showing the gross features of the implant with bone attachment throughout the surface of the implant and cement surrounding the crown edges; (**b**) discoloration is also evident in this example; the smooth surface was covered by cement; (**c**) implant rough interface with evidence of crack development. The arrows highlight a large pit with crack nucleation inside; and (**d**) higher magnification of an exposed area of the abutment with scratching.

Implant 5 had to be subjected to extensive cleaning, due to biological deposition, bone attachment in the surface and cement surrounding the smooth collar areas (Figure 6a). The typical surface discoloration observed with the other specimens was also visible with this implant (Figure 6b) post-cleaning. High magnification microscopy of the rough interfaces revealed the presence of pitting attack and branched cracks (Figure 6c), similar to the crack pattern observed with implant 4. A large pit (diameter > 20 μm) is highlighted in Figure 6c, illustrating crack nucleating inside the feature. The exposed areas of the abutment also revealed pitting attack and severe scratching. EDS analysis of both the smooth collar (areas not covered by cement) and rough interfaces of this implant provided the presence of very high percentages of Ti (~65%–89%), with lower concentrations of the same element (~55%) in areas where biological deposits were not removed with cleaning. A summary of the events observed on the surface of the implants is shown in Table 2.

**Table 2:** Summary of events observed on the surface of the retrieved titanium dental implants.

Implant ID	Visual Inspection	Morphology (Optical/SEM)	Composition (Mass %)	Corrosion Mechanisms
Control sample	Clean interfaces	No defects Few scratches on abutment	Ti: 87%–92% O: 3%–5% O C: 4%–6% N: 1%–2%	None
Implant #1	Rust Discoloration	Etching Microstructural attack Pitting attack mostly in the smooth surfaces of the abutment Scratching of the abutment	Ti: 50%–60% O: 13%–32% C: 23%–48% N: 3%–6% P: 2%–5% Ca: 3%–11% Zr: 0.7%–3% S: 0.2%–0.3%	Tribocorrosion (fretting and electrochemistry)
Implant #2	Rust Discoloration	Etching Microstructural attack Pitting attack in areas with discoloration Cracking Delamination	Ti: 60%–80% O: 12%–28% C: 7%–22% N: 2%–6% P: 1%–2% Ca: 0.4%–4% S: 0.05%–0.3%	Tribocorrosion
Implant #3	Bone attached Dents Scratches	Etching Microstructural attack Severe pitting attack in the abutment and rough surfaces Scratching	Ti: 50%–60% O: 4%–21% C: 17%–25% N: 5% Ca: 3%–10% Al: 5%–10% V: 2%–4%	Tribocorrosion
Implant #4	Rust Discoloration Biological deposits	Etching Severe scratching Pitting attack in the rough interfaces and abutment areas Deformation Bulk exposure	Ti: 12%–70% O: 7%–30% C: 13%–41% N: 3% Ca: 2%–10% Nb: 7% Al: 5% P: 6%	Tribocorrosion
Implant #5	Bone and cement deposition	Pitting attack on the smooth and rough interfaces Scratching Discoloration	Ti: 60%–85% O: 4%–14% C: 4%–22% N: 3%–12% Ca: 1%–2% V: 1%–1.5%	Tribocorrosion

## 4. Discussion

The analysis of the five retrieved (Ti) dental implants revealed cases of severe surface damage that appeared to be the result of a synergistic mechanism between chemical attack and mechanically-induced degradation (tribocorrosion). Typical and common features observed among the implants evaluated included surface etching, pitting attack of the smooth collar areas of the implant and, rough interfaces, severe scratching of the abutment-crown interfaces and cracking of the implant rough surface. These observations led to the hypothesis that the chemical attack was produced by significant drops in pH and that the synergy between these chemical and mechanical mechanisms led to a process of fretting-crevice-corrosion.

Bacteria present in the oral environment and/or forming biofilm on the implant surface are expected to reduce the pH surrounding a dental implant to the levels required to trigger oxidation and Ti dissolution [2,3,4]. Inflammatory processes can also contribute to enhancing the acidity of the medium. The analysis of implant 4 (Figure 5), for example, demonstrated a case of severe bulk attack with signs of pitting and discoloration. This particular implant was removed after only four weeks of implantation, due to signs of inflammation, which led to the conclusion that a very acidic environment was probably triggered by bacteria associated with the inflammatory process. The highly acidic medium induced active dissolution of the bulk, which, in combination with normal mastication loads, led to permanent disruption of the oxide film and active attack of the underlying bulk metal. Another sign that confirmed that this particular implant was subjected to a highly acidic pH was the violet and yellow discoloration of the interfaces of the implant. Similar characteristics were observed with all of the other implants analyzed. The lack of clinical information for the other four implants limits conclusions regarding the possibility of bacteria or inflammation promoting a similar acidic environment.

Analysis of each individual implant revealed several common mechanisms, which may be an indication of the most prevalent failure modes with Ti dental implants. Implant 1 (Figure 2) exhibited severe pitting attack of the smooth collar portions of the implant, which is an area of high exposure to the biological environment. Scratching was also evident, indicating that a mechanism of fretting-crevice corrosion was present. The degree of surface damage leads us to conclude that a large amount of particle debris and ions was generated from the pitted and scratched areas of this implant. Excessive micromotion, due to normal cyclic occlusal forces, in combination with a highly acidic environment, were likely responsible for the failure of this particular implant. Implants 2, 4 and 5 (Figure 3, Figure 4 and Figure 6, respectively) had different designs, sizes and material compositions (commercially pure titanium, cpTi, *versus* alloy); however, the surface conditions of these three implants were very similar. A characteristic feature was the discoloration of the rough interfaces, as observed with implant 1. This again is a strong indication of a highly acidic environment. All of these implants also exhibited pitting attack and scratching of the smooth and exposed interfaces of the implant. Because the crown was still in place with implants 2 and 5, it was not possible to access the degree of damage internally on the abutment. Because the crown-abutment couple forms a crevice, this area of the implant is particularly susceptible to fretting-crevice corrosion. Implant 3 (Figure 4) also exhibited a case of severe delamination of the smooth and exposed interfaces of the abutment. This was confirmed by EDS results, which showed very high concentration of Ti. Delamination to such an extent will lead to the generation of large pieces of metal in the surroundings of the implant, which can accumulate in the adjacent tissues. Pitting attack was also prevalent in the rough interfaces of this implant. Another interesting feature with these implants was evidence of cracking of the rough interfaces (Figure 3c, Figure 5c and Figure 6c). The cracks seem to have nucleated inside pits with mostly branched patterns, which is an indicator of stress-corrosion processes [17]. In some cases, the cracks showed some depth and were filled with biological matter (Figure 5c), which also indicates that a large concentration of particles and metal ions could have been generated *in vivo.* Implant 3 had the same characteristic discoloration and severe pitting attack on the smooth and exposed interfaces of the abutment. Pitting attack was also prevalent on the implant rough interfaces with deformation and delamination.

In all the specimens analyzed, the EDS of corroded areas (Table 2) revealed a predominant signal of C, instead of Ti or O, which were predominant in areas where the surface had no signs of corrosion or other mechanisms of failure. In areas with cracking, pitting and bulk exposure, Ti was predominant, with lower concentrations of O, indicating permanent breakdown of the oxide film. The Ti discoloration observed with the set of implants has been reported in other retrieval studies involving failed hip implants [9]. Furthermore, this characteristic discoloration confirms that these implants were indeed exposed to a very acidic environment. Sartori *et al.* [18] observed that when the pH of the testing medium was very low, stains and dark spots were apparent on Ti specimens surveyed under SEM. Gilbert *et al.* [8] demonstrated in a retrieval study of modular hip implants that the pH can drop to values as low as 1in Ti crevice areas, which caused dissolution and precipitation of Ti phosphate particles (Ti^3+^) with a characteristic violet discoloration. Bhola *et al.* [19] reported that in strong reducing acids, the unprotected bulk metal is oxidized to the violet colored trivalent ion (Ti^3+^), which can be further converted to the pale yellow Ti^4+^ ion. Whether the pH drop was caused by bacteria could be only confirmed if the complete clinical history of the implants was known. Regardless, these observations support the hypothesis that bacteria responsible for causing peri-implantitis or other inflammatory processes can facilitate metal attack.

Pitting attack on the abutment, smooth, and exposed interfaces was also present in the majority of the cases evaluated. Again, this scenario raises the possibility of bacteria contributing to corrosion, since this area of the implant is highly exposed to the dental environment. Pitting attack leads to accelerated and localized metal dissolution, which will contribute to the metal ion and particle burden surrounding the implant and tissues. EDS analysis of areas exposed to pitting attack revealed the presence of sulfur (S). Sulfur-containing metabolites, such as the redox-active amino acid, cysteine, may be liberated during bacterial metabolism, hence the possibility of a bacterial biofilm triggering the oxidation. Pitting attack has been previously observed with Ti6Al4V hip implants exhibiting signs of severe corrosion [9]. Cheng *et al.* [20] demonstrated with SEM analysis the presence of pitting attack of Ti in fluorinated solutions. Contrary to these observations, Bhola *et al.* [19] suggested that pitting corrosion of Ti is not of much concern in the oral environment, because Ti oxide films induce a very high anodic pitting potential, which gives immunity to pitting. The present study provides evidence that Ti dental implants are highly susceptible to pitting attack in the oral environment.

The abutment-crown interface is another area of the implant that can be subjected to fretting crevice corrosion, due to the modular nature of the interface. However, fretting-crevice corrosion can also be induced on the rough interfaces of the implant in the event a bacterial biofilm is formed and adheresto the surface. This scenario would further contribute to the acidity of the environment, especially if biological fluids get entrapped between the implant surface and the biofilm. In the restricted area of a crevice, depletion of oxygen supply will turn the medium extremely acidic, which will incur accelerated oxidation. The pitting observed on the rough interfaces of the implant could have been a result of a similar mechanism. In cement-retained restorations, there is also the possibility of the cement inducing changes in the contacting metal abutment. Wadhwani *et al.* [20] reported that a polycarboxylate cement resulted in pitting corrosion when in contact with Ti6Al4V.

The degradation observed with these implants leads us to conclude that a large amount of particle ions and debris was generated *in vivo* and may have contributed to the clinical implant failure. Titanium and its alloys are known for having excellent biocompatibility and corrosion resistance; however, Ti metal ion release has been previously reported *in vivo* and with *in vitro* experiments [21]. Kumazawa *et al.* [22] reported that Ti particles can indeed induce a cytotoxic response *in vivo*. Recent studies in the orthopedic literature, which evaluated the surface of retrieved hip implants and adjacent tissues, also demonstrated an adverse tissue response to metal ions released from metal-on-metal interfaces [23]. Characterization of failure mechanisms with these implants is crucial to provide evidence in relation to their trends, location or nature [24]. It has been demonstrated in this study that events, such as delamination, pitting attack and cracking, illustrate cases in which the passivity of the metal was disrupted. These events are assumed to have led to the dissolution of the underlying metal, inducing a large amount of metal ions and debris in the surrounding tissues. While it is not possible to conclude that corrosion products and metallic debris from these implants led to an inflammatory response or accelerated a pre-existing condition (peri-implantitis, peri-implant mucositis),because of the lack of clinical data, these observations give clear indication that severe degradation of Ti dental implants can be induced*.*

It is important to note that not only bacteria, potent inflammatory mediators, or implant overloading can be the triggering factors for oxidation, but also under normal circumstances, it has been shown that the passivity of dental alloys can be disrupted in the presence of solutions that can turn the oral environment acidic (fluoride, hydrogen peroxide, *etc.*) [19,20,25,26]. Oral hygiene products containing fluoride have been reported to detrimentally affect the properties of Ti alloys. Literature reports showed that sodium-fluoride prophylactic gels in concentrations higher than 0.1% can affect the passivity of Ti and its alloys in the oral environment [27,28,29]. Considering the fact that most implant patients use prophylactic fluoride-containing gels, dentifrices or supplementary fluoride, it is possible that fluoride ions contributed to the observed degradation with the implants evaluated at accessible sites.

Based on this study and previous observations, it is evident that the synergy between implant micromotion and acidic environment can permanently break down the titanium oxide barrier against metallic corrosion. This phenomenon can affect the integration of the implant with native tissue, causing loss of the implant [6]. Understanding the triggering mechanisms for corrosion in the oral environment and finding ways to mitigate degradation processes *in vivo* is of great importance. Additional evidence is also needed to support the hypothesis that corrosion can be one of the main factors inducing peri-implantitis. Corrosion has been regarded in the literature as a secondary triggering factor in inducing inflammation and peri-implantitis [6], and the lack of studies relating peri-implantitis to Ti corrosion makes it difficult to reach any further conclusions. Mouhyi *et al.* [6] suggested that peri-implantitis seems, however, to be associated with disappearance of the titanium oxide layer (TiO_2_). This may be a fact, given that the mechanisms reported in the present study clearly show areas of the implant with bulk exposure, a result of the disappearance of the Ti oxide film. Olmedo *et al*. [26] and Bhola *et al*. [19] made a related point, speculating that corrosion-induced ions could be associated with peri-implantitis. The rationale was that leached particles could be phagocytosed by macrophages and release mediators of inflammation, which could potentially inhibit osteoblast formation.

Further investigation is needed to identify other potential mechanisms of dental implant degradation and their relation to peri-implantitis. The observations of this study indicate that metal ions and debris originating from corrosion processes may promote adverse tissue reactions that could lead to peri-implantitis. A better understanding of the synergistic and individual effects of bacterial biofilm and implant loading on the surface of a dental implant is needed to fully appreciate all the contributing factors in the failure process. Addressing all these questions is critical for enabling preventive measurements for the accumulation of pathogenic bacteria on the surface of dental implants and mitigation of corrosive processes.

## 5. Conclusions

Surface analysis of five retrieved dental implants showed features that give an indication of the presence of acidic conditions, which triggered metal oxidation in the oral environment. Events, such as scratches, pitting, fretting-crevice corrosion, cracks and surface delamination, were present in all of the specimens evaluated. The specimens also exhibited a characteristic surface discoloration, which results when unprotected bulk titanium is oxidized to the violet-colored trivalent ion (Ti^3+^). SEM/EDS analysis corroborated bulk metal ion dissolution with evidence of regions rich in Ti and with complete depletion of the Ti oxide film. Corrosion products and metal ions resulting from metal dissolution can lead to adverse tissue reactions in the oral environment. Moreover, osseointegration may not happen, once surface passivation is lost. The observed chemical attack was produced by a significant drop in pH, which was potentially induced by bacteria. The conjoint effects of electrochemical factors, bacterial biofilm and implant loading can lead to a severe degradation process *in vivo*, which can trigger or further accelerate peri-implantitis or other diseases that can result in implant failure.
